# An Overview on Nutritional Aspects of Plant-Based Beverages Used as Substitutes for Cow’s Milk

**DOI:** 10.3390/nu13082650

**Published:** 2021-07-30

**Authors:** Isabel Fructuoso, Bernardo Romão, Heesup Han, António Raposo, Antonio Ariza-Montes, Luis Araya-Castillo, Renata Puppin Zandonadi

**Affiliations:** 1 Department of Nutrition, University of Brasília, Brasília 70910-900, Brazil; belfructuoso@gmail.com (I.F.); bernardolima156@gmail.com (B.R.); renatapz@unb.br (R.P.Z.); 2 College of Hospitality and Tourism Management, Sejong University, 98 Gunja-dong, Gwanjin-gu, Seoul 143-747, Korea; 3 CBIOS (Research Center for Biosciences and Health Technologies), Universidade Lusófona de Humanidades e Tecnologias, Campo Grande 376, 1749-024 Lisboa, Portugal; 4 Social Matters Research Group, Universidad Loyola Andalucía, C/Escritor Castilla Aguayo, 4 14004 Córdoba, Spain; ariza@uloyola.es; 5 Facultad de Economía y Negocios, Universidad Andrés Bello, Santiago de Chile 7591538, Chile; luis.araya@unab.cl

**Keywords:** plant-based beverage, nutritional compositional, added ingredients, energy, macronutrients, dietary fiber, micronutrients

## Abstract

The presence of milk in meals and products consumed daily is common and at the same time the adoption of a milk-free diet increases due to milk allergy, lactose intolerance, vegan diets, and others. Therefore, there is an increasing demand for plant-based beverages, which present variable and, sometimes, unknown nutritional characteristics. This study sought to compare the nutritional aspects of plant-based beverages used as substitutes for cow’s milk described in scientific studies. Therefore, we used a review of the scientific literature on PubMed, Google Scholar, Scopus, Web of Science, Google Patents, Embase, and ScienceDirect databases. The inclusion criteria were scientific studies referring to plant-based beverage used as an alternative to cow’s milk; published in the English language; present data on the serving size, ingredients, and nutritional composition, containing at least data on energy and macronutrients of plant-based beverages. Ingredients and data on energy, macronutrients, and, if available, dietary fiber and some micronutrients of plant-based beverages were collected. Data were obtained from 122 beverages of 22 different matrices, with soy being the most used (27.87%, *n* = 34). The variation in the amount of nutrients found was 6–183 Kcal/100 mL for energy value; 0.00–22.29 g/100 mL for carbohydrate; 0.06–12.43 g/100 mL for protein; 0.00–19.00 g/100 mL for lipid; 0.00–4.40 g/100 mL for dietary fiber; 0.00–1252.94 mg/100 mL for calcium; 0.04–1.40 mg/100 mL for iron; 0.84–10,178.60 mg/100 mL for magnesium; 0.00–343.43 mg/100 mL for sodium. Salt was the most commonly found added ingredient in plant-based beverages. Some beverages have reached certain amounts of cow’s milk nutrients. However, studies have pointed out differences in their qualities/types. Thus, attention is needed when replacing milk with these alternatives.

## 1. Introduction

Milk is the “lacteal secretion, practically free from colostrum, obtained by the complete milking of one or more healthy cows” [1]. Worldwide, cow’s milk and its derivatives are consumed by more than 6 billion people [2], on average of 116.50 kg/inhabitant/year [3], standing out mainly due to its content of high biological value proteins and calcium [4,5,6].

Despite the dietary benefits provided by milk, some people present milk-related disorders as cow’s milk allergy (CMA), which reaches about 0.50% and 3.50% of individuals [6,7,8], and lactose intolerance (65.00–75.00% of individuals) [6,9,10,11,12]. Additionally, some people choose to follow a milk-free diet like some types of vegetarians and vegans [13]. Given the high presence of milk in meals and products consumed daily and people who follow a milk-free diet, it is necessary to seek alternatives to replace this product in daily meals [14].

Regarding CMA, some animal-based alternatives may be used to replace cow’s milk, such as donkey and goat milk [15,16,17]. Nevertheless, an altered version of cow’s milk, the A2A2 kind where cow’s milk main protein A1 β-casein is modified to a non-allergenic version, the A2 β-casein type, can also be used as an alternative with good results [18]. Though, despite the replacement of the main animal protein, these options often present lactose, which may be an impairment to a considerable part of the population [12].

Considering all milk-related disorders, the main alternatives used to replace milk are water-soluble extracts of legumes (e.g., soybean (*Glycine max*) and chickpea (*Cicer arietinum*)), nuts (e.g., almond (*Prunus dulcis*), cashew nut (*Anacardium occidentale*), hazelnut *Corylus avellana*), and Brazil nut (*Bertholletia excelsa*), seeds (e.g., sunflower (*Helianthus annus*) and sesame (*Sesamum indicum*)), cereals (e.g., rice (*Oryza* spp.) and oat (*Avena sativa*)) or pseudocereals (e.g., quinoa (*Chenopodium quinoa*)) [5,19,20,21]. It is important to highlight that consumers search for a product to be used as cow’s milk substitute with similar sensorial aspects regarding color, texture and, when possible, flavor [22]. However, frequently, milk substitutes’ nutritional characteristics are not similar to cow’s milk [10].

Plant-based beverages present the composition variable on the amount of macro and micronutrients and the presence of bioactive compounds and antinutritional factors [10]. As for bioactive compounds, for example, the soy-based beverage contains isoflavones and phytosterols; almond-based beverage contains α-tocopherol and arabinose; oat-based beverage contains β-glucan. Regarding antinutritional factors, for example, there is the presence of oxalates and phytates in the sesame-based beverage and phytates in the oat-based beverage. Technological interventions are being studied to improve these beverages’ sensory acceptability and nutritional quality [10,19]. Despite that, we hypothesized that plant-based beverages are lower in protein and calcium than cow’s milk, but some of them are better than others regarding nutritional quality.

The demand for plant-based beverages has been increasing worldwide over the years [6,10,11,14,23,24,25] due to their potential health benefits [14,19] and the growing adoption of a milk-free diet. It is estimated an annual growth rate of 10.18% between 2020 and 2024 [26].

Considering the scarcity of studies comparing the nutritional composition of different milk substitutes to guide the population about the best alternative to compose their diet, this study sought to compare the nutritional aspects of plant-based beverages used as substitutes for cow’s milk described in scientific studies.

## 2. Materials and Methods

### 2.1. Data Collection

Data were collected on plant-based beverages from publications in scientific journals without the geographical limitations of the studies. PubMed, Google Scholar, Scopus, Web of Science, Google Patents, Embase, and ScienceDirect databases were searched and the search terms used are available in Appendix A.

#### 2.1.1. Inclusion and Exclusion Criteria

The inclusion criteria were scientific studies referring to plant-based beverage used as an alternative to cow’s milk; have been published in English; present data on the serving size, ingredients, and nutritional composition, containing at least data on energy (kcal) and macronutrients (carbohydrates, protein, and lipids) of plant-based beverages.

#### 2.1.2. Collected Information

The data collected from scientific studies were authors; country of origin; year of publication; types of plant-based beverages presented; serving size of the beverages analyzed; nutritional composition; ingredients; specifications on the origin of the beverages, that is, if the beverage was prepared and analyzed for the study or if it is a beverage available on the market and had its nutritional values removed from the label, among other information. Regarding nutritional composition, the values collected from plant-based beverages were energy value; macronutrients (carbohydrates, protein, and lipids); if available, dietary fiber, calcium, iron, magnesium, and sodium.

The nutrient values were converted to the equivalent of 100 mL using Microsoft Excel^®^. For the serving sizes in grams, a density of 1.027 g/mL was considered, based on the soy-based beverage values, and the calculations were made [27]. When available, salt values were converted into the equivalent in sodium, given that 1 g of salt presents 400 mg of sodium [28]. Microsoft Excel^®^ was used to standardize data.

### 2.2. Data Processing

With the worldclouds platform’s help, a word cloud was generated with the ingredients excluding water and the beverages’ implemented matrix, for example, “almond” [29]. Names were represented according to their proportional frequencies among all ingredients in all analyzed samples, given that higher frequencies result in words with bigger sizes.

## 3. Results

A total of 32 studies were selected according to the inclusion and exclusion criteria. The studies were conducted in 13 different countries: Brazil (59.38%; *n* = 19) [30,31,32,33,34,35,36,37,38,39,40,41,42,43,44,45,46,47,48]; India (6.25%; *n* = 2) [49,50]; Argentina (3.13%; *n* = 1) [51]; Belgium (3.13%; *n* = 1) [52]; Canada (3.13%; *n* = 1) [23]; Denmark (3.13%; *n* = 1) [53]; Germany (3.13%; *n* = 1) [54]; Ghana (3.13%; *n* = 1) [55]; Iran (3.13%; *n* = 1) [56]; Ireland (3.13%; *n* = 1) [57]; Nigeria (3.13%; *n* = 1) [58]; Pakistan (3.13%; *n* = 1) [59]; Turkey (3.13%; *n* = 1) [60].

From these studies, data were obtained from 122 beverages made from 22 different matrices. Of these matrices, 18.18% (*n* = 4) belong to the group of cereals that constituted a total of 18.85% (*n* = 23) of beverages: rice (*Oryza* spp.) (11.48%, *n* = 14) [30,37,46,52,53,54,57,60]; oat (*Avena sativa)* (5.74%, *n* = 7) [31,50,52,53,54,57]; millet (*Setaria italica*) (0.82%, *n* = 1) [54]; spelt (*Triticum spelta*) (0.82%, *n* = 1) [54].

Concerning legumes, 13.64% (*n* = 3) of the matrices are part of this group, totaling 29.51% (*n* = 36) beverages: soy (*Glycine max*) (27.87%, *n* = 34) [23,38,42,43,44,48,52,53,54,55,56]; *baru* almond (*Dipteryx alata*) (0.82%, *n* = 1) [40]; groundnut (*Arachis hypogaea*) (0.82%, *n* = 1) [49].

A total of 36.36% (*n* = 8) of the matrices belong to the group of nuts that constituted 41.80% (*n* = 51) of beverages: almond (*Prunus dulcis*) (9.84%, *n* = 12) [23,36,52,53,54,57,58]; *sapucaia* nut (*Lecythis pisonis*) (9.02%, *n* = 11) [47]; cashew nut (*Anacardium occidentale*) (6.56%, *n* = 8) [23,32,33,52,54,57,59]; coconut (*Cocos nucifera*) (6.56%, *n* = 8) [23,52,53,54,57]; hazelnut (*Corylus avellana*) (3.28%, *n* = 4) [53,54,57]; *cupuaçu* almond (*Theobroma grandiflorum*) (2.46%, *n* = 3) [35]; *tucumã* almond (*Astrocaryum vulgare*) (2.46%, *n* = 3) [35]; macadamia nut (*Macadamia* spp.) (1.64%, *n* = 2) [54,57].

About pseudocereals, 9.09% (*n* = 2) of the matrices are part of this group that were used to produce a total of 3.28% (*n* = 4) of beverages: quinoa (*Chenopodium quinoa*) (2.46%, *n* = 3) [39,53,57]; amaranth (*Amaranthus hypochondriacus*) (0.82%, *n* = 1) [51]. Regarding seeds, 4 (18.18%) of the matrices correspond to this group that generated a total of 5.74% (*n* = 7) of beverages: hemp (*Cannabis sativa*) (2.46%, *n* = 3) [23,53,57]; sesame seed (*Sesamum indicum*) (1.64%, *n* = 2) [34]; safflower seed (*Carthamus tinctorius*) (0.82%, *n* = 1) [49]; sunflower seed (*Helianthus annus*) (0.82%, *n* = 1) [45]. In the case of tubers, only 4.55% (*n* = 1) of the matrices represents this group, being the yam (*Dioscorea* spp.) that constituted 0.82% (*n* = 1) of beverages [41].

Regarding the origin of the 122 beverages and how the nutritional data were obtained, 54.92% (*n* = 67) of the beverages were prepared for the study, of which 50.82% (*n* = 62) were analyzed to determine their nutritional composition [31,32,33,34,35,36,37,38,39,40,42,43,44,45,46,47,48,49,50,51,55,56,58,59,60]; 3.28% (*n* = 4) had their nutritional values determined based on the label of the product of origin [30]; 0.82% (*n* = 1) had their nutritional data taken from Brazilian Nutritional Composition Table and Tables of Nutritional Composition of Foods Consumed in Brazil of IBGE (*Instituto Brasileiro de Geografia e Estatística*) [41,61,62].

Considering other beverages, 32.79% (*n* = 40) were commercial plant-based beverages used as alternatives for cow’s milk from different brands whose nutritional values were obtained from the labels [52,53,54,57]; 12.30% (*n* = 15) had their nutritional composition evaluated using USDA (United States Department of Agriculture) Food Composition Database [23].

Table 1 shows the nutritional composition, ingredients and specifications on the origin of the beverages. The identification information (authors, year of publication and country of origin) collected from the studies is presented in Appendix B.

Considering Table 1, the energy value ranged from 6 to 183 Kcal/100 mL, the carbohydrate content ranged from 0.00 to 22.29 g/100 mL, the protein content ranged from 0.06 to 12.43 g/100 mL, and the lipid content ranged from 0.00 to 19.00 g/100 mL. The most caloric beverage collected and with the highest lipid content is the coconut-based beverage from the Real Thai brand [54]. Four rice-based beverages of different types (parboiled, brown, polished and red) presented the lowest lipid value [30].

The least caloric and with the lowest protein content is the *tucumã* almond-based beverage extracted at a temperature of 55 °C [35]. The beverage collected with the highest protein content is the concentrated fluid from stage 5 of the crioconcentration process that the *sapucaia* nut-based beverage was submitted to [47].

Regarding carbohydrates, the sesame-based beverage that contains maltodextrin as an ingredient has the highest carbohydrate content [34], while the sunflower seed-based has the lowest carbohydrate content [45]. Among the beverages collected that dietary fiber values were provided for (18.03%, *n* = 22), this nutrient content ranged from 0.00 to 4.40 g/100 mL. An oat-based beverage has the highest dietary fiber content [50], while three rice-based beverages of parboiled, polished and red rice types [30], and a coconut-based beverage [52] have the lowest dietary fiber value.

With regard to the collected micronutrients, the amount of beverages that provided these data were 44.26% (*n* = 54) for calcium, 6.56% (*n* = 8) for iron, 13.11% (*n* = 16) for magnesium, and 22.95% (*n* = 28) for sodium. The calcium content ranged from 0.00 to 1252.94 mg/100 mL, iron content ranged from 0.04 to 1.40 mg/100 mL, magnesium content ranged from 0.84 to 10,178.60 mg/100 mL, and sodium content ranged from 0.00 to 343.43 mg/100 mL.

The concentrated fluid from stage 5 of the crioconcentration process that the *sapucaia* nut-based beverage was submitted has a higher content of calcium, magnesium, and sodium [47]. An almond-based beverage has the highest content of iron [58].

Three rice-based beverages (with brown, parboiled and red rice types) [30], one soy-based beverage and two coconut-based beverages [23] have the lowest calcium content. A rice-based beverage made with broken polished rice has the lowest content of iron and magnesium [46]. Considering sodium, three rice-based beverages (with brown, polished and red rice types) presented the lowest sodium content [30].

**Figure 1:**
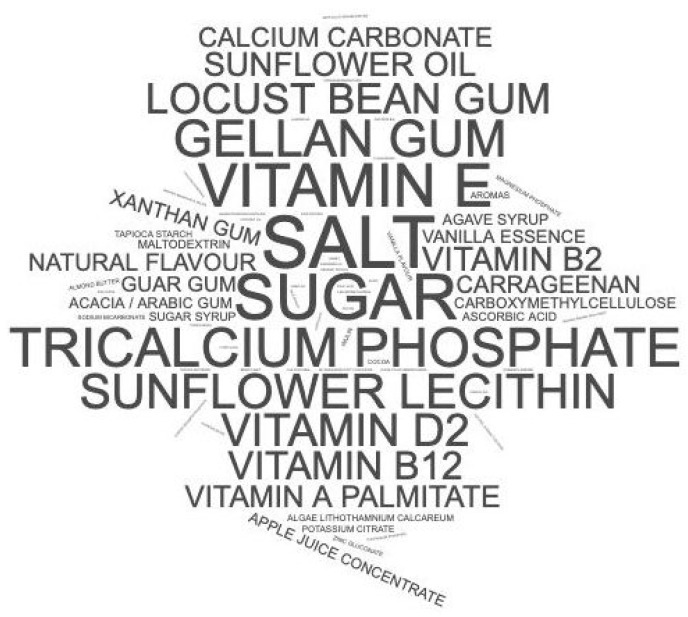
Word cloud of added ingredients from plant-based beverages from scientific studies selected according to the inclusion and exclusion criteria. Words are presented according to their proportional frequencies among all samples, given that greater sizes correspond to greater frequencies.

Excluding water and the matrix from the beverages, the most commonly found ingredients in plant-based beverages were salt (50 times), sugar (21 times), vitamin E (tocopherol) (20 times), tricalcium phosphate (18 times) and gellan gum (17 times) (Figure 1).

## 4. Discussion

### 4.1. Nutritional Content Variations

This review confirmed that plant-based beverages’ nutritional content depends on different aspects, such as the type of raw material used to produce it, the process, and the added ingredients [11,21].

#### 4.1.1. Processing Performed

The *sapucaia* nut-based beverage (the *sapucaia* nut (*Lecythis pisonis*) is a Brazilian species found in the Amazon and Atlantic Forest) was subjected to crioconcentration in block (method of total freezing followed by partial thawing of the solution, under gravitational separation), capable of concentrating solid matter by removing water in ice form [47]. This process concentrates nutritional compounds, such as protein, carbohydrate, calcium, and magnesium, influencing the beverage’s nutritional content [47]. This concentrated water-soluble extract can be used as an ingredient for other food products to improve their nutritional content as selenium, in which the concentrated extract could reach 10,037 μg/g [47].

The hot grinding method—capable of inactivating the lipoxygenase enzyme of the water-soluble soy extract that causes its typical “beany” flavor—[63] and the cold grinding method (traditional oriental method) were performed to obtain water-soluble soy extract [44]. The cold-extracted beverages had a higher moisture content and, consequently, lower concentrations of proteins and total solids than the hot-extracted beverages [44].

A study analyzed the effect of physical and antioxidant treatments on the lipoxygenase enzyme activity in a soy-based beverage [43]. The author observed that the protein and lipid content of BRS-213 cultivar’s beverages subjected to physical treatments (irradiation—5.00 kGy; thermal—80 °C) with tocopherol supplementation were higher than the control sample. The study showed that adequate heat treatment increases lipids and proteins content, as well as their solubility and digestibility [43].

The physical treatments (irradiation—5.00 kGy; thermal—80 °C) with tocopherol supplementation in BRS-213 cultivar’s beverages also increased the macronutrient concentration compared to the control sample due to lower moisture content [43]. Furthermore, the beverages produced with the EMB-48 soy cultivar subjected to heat treatment had a significant increase in lipid and protein content [43]. The hydrothermal processing of the soy-based beverage caused a rupture in the soy cell wall, allowing the passage of lipids and proteins, which was responsible for increasing the content of these nutrients [43].

As for irradiation (conservation technique) to which soy-based beverages were subjected, a study [42] verified a significant reduction in the protein content when increasing the dose of gamma radiation. Deamination (release of the amine group from the rupture of the peptide bonds of the amino acid, forming ammonia) was mentioned as a possible cause [42]. However, this process may have improved the protein quality since deamination makes it more hydrophilic, increasing its solubility and digestibility [42].

Another aspect in producing plant-based beverages capable of influencing its nutritional content is the raw material:water ratio. A study [48] found higher macronutrient and energy concentration in the soy-based beverage with a 1:8 ratio (soy:water) than in those with a 1:10 and 1:12 ratio. These results were expected since water dilution implies nutrients dilution in the final product.

#### 4.1.2. Added Ingredients

In the production of plant-based beverages, the addition of other ingredients may improve the nutritional profile, the functional or sensory properties of the beverage [22,64]. Salt, sugar, syrups, flavorings, vanilla essence, cocoa, apple juice concentrated, and others are incorporated to improve sensory aspects, mainly the flavor [42,64]. In addition to enhancing flavor, salt and sugar are usually added in beverages and foods to improve texture or shelf-life [65].

Cow’s milk presents a mild and typical flavor, neither sweet nor salty, characterized mainly by the balance between lactose and salt [4]. Therefore, it is common to find salt in plant-based beverages, as well as ingredients for a sweet flavor (sugar, maltodextrin, apple juice, agave syrup, vanilla extract). It is important to highlight that the use of ingredients like sucrose, maltodextrin, and fructose might negatively impact the glycemic index and salt also might impair the nutritional quality. The consumption of foods with low (<55) and medium (56–69) glycemic index is recommended, especially to control blood glucose levels [20,23]. A low glycemic load diet is related to a lower risk of diabetes, cardiovascular disease, and obesity [66].

Sugar is often used to improve sensory quality but influences the nutritional quality negatively. In the studies by Barros and Venturini Filho [44] and Karimidastjerd and Kilic-Akyilmaz [60], the plant-based beverages with added sugar are more caloric and with higher amounts of carbohydrate than those without added sugar. In the sesame-based beverages studied by Reis [34], the carbohydrate amount of the sample added with maltodextrin is almost twice the amount of the pure sesame-based beverage. Consequently, the sample with maltodextrin is more caloric [34].

Other ingredients commonly found in plant-based beverages are vegetable oils, such as sunflower oil. Martínez-Padilla et al. [53] pointed out that the addition of these oils can provide a smooth mouthfeel similar to that of cow’s milk, and Aydar et al. [64] mentioned its use in order to improve the silky aspect.

A study [22] showed that it is possible to add starch in plant-based beverages as a plant-derived thickening agent and pectin or locust bean gum to improve the texture. Reis [34] used maltodextrin to improve the stability of the beverage and Sethi et al. [19] referred to the use of sodium bicarbonate as an alkalizing agent, which might prevent destabilization by sedimentation of solid particles in the beverage.

A study [39] used enzymes in the quinoa-based beverage preparation, with the addition of the Termamyl enzyme for the dextrinization process and the amyloglucosidase enzyme for saccharification. In food industry, inulin can be used as a texture modifier, sugar or fat substitute, and it also has a prebiotic effect on the human organism [67].

Due to the nutritional composition of most plant-based beverages (not similar to that of cow’s milk—high in protein and calcium) and nutritional losses during the processing of the raw material, in plant-based beverages the addition of vitamins (e.g., A, D, E, B2, folic acid, B12), minerals (e.g., calcium, zinc) and proteins (usually isolated or extracted from sources, for example, peas or soy) is frequent, trying to achieve a similar composition to cow’s milk [68]. However, other vitamins and minerals are added with different functions. Barros [43] added tocopherol (vitamin E) with the function of antioxidant treatment of beverages.

Zinc gluconate, zinc oxide, calcium carbonate, tricalcium phosphate and monocalcium phosphate are some of the micronutrient compounds used as food fortifiers [69]. Algae *Lithothamnium calcareum* is mentioned by Scholz-Ahrens et al. [54] as another source of calcium added to plant-based beverages. Although these beverages are often fortified, the added nutrients are not always bioavailable as those naturally present in milk [68].

Tricalcium phosphate can be used for several functions as an acidity regulator, buffer, anticaking agent, clouding agent, emulsifying, firming agent, flour treatment agent, humectant, moisture-retention agent, raising agent, stabilizer and texturizing agent; calcium carbonate can be used as an acidity regulator, anticaking agent, surface colorant, firming agent, dough conditioner, and stabilizer [70]. Some of these functions are important in plant-based beverages, but they were not discussed in the studies that use them.

The function of gums as food additives can also be used in plant-based beverages. In this sense, gellan gum is used as a gelling agent, stabilizer, and thickener; locust (or carob) bean gum and guar gum are used as an emulsifier, stabilizer, and thickener; xanthan gum as an emulsifier, foaming agent, stabilizer and thickener; gum arabic (acacia gum) is used as a bulking agent, carrier, emulsifier, glazing agent, stabilizer and thickener [70].

The technological purposes of other food additives that appeared in the beverages ingredients lists are carrageenan (bulking agent, carrier, emulsifier, gelling agent, glazing agent, coating agent, humectant, stabilizer and thickener); lecithin (antioxidant and emulsifier); ascorbic acid (acidity regulator, antioxidant, flour treatment agent and sequestrant); citric acid (acidity regulator, antioxidant, color retention agent, and sequestrant); sodium carboxymethyl cellulose (bulking agent, emulsifier, suspension agent, firming agent, gelling agent, glazing agent, coating agent, humectant, stabilizer and thickener); sodium potassium hexametaphosphate (acidity regulator; emulsifier, moisture-retention agent, raising agent, sequestrant, stabilizer and texturizing agent); sodium metabisulfite (antioxidant, bleaching agent, flour treatment agent and preservative); potassium citrate (acidity regulator, emulsifying salt, sequestrant and stabilizer); potassium phosphate (acidity regulator, buffer, emulsifier, emulsifying salt, humectant, moisture-retention agent, sequestrant, stabilizer and texturizing agent); magnesium phosphate (acidity regulator, anticaking agent, emulsifying salt, raising agent, stabilizer and thickener); sucrose esters of fatty acids (emulsifier, foaming agent, glazing agent and stabilizer); mono- and di-glycerides of fatty acids (antifoaming agent, emulsifier, glazing agent, surface-finishing agent and stabilizer) [70].

### 4.2. Comparison of Nutritional Composition: Plant-Based Beverages and Cow’s Milk

As a reference for comparing the nutritional composition of the types of plant-based beverages, the nutritional composition (per 100 g) of a whole cow’s milk obtained from USDA are energy (64 Kcal), carbohydrate (4.65 g), protein (3.28 g), lipid (3.66 g), calcium (119.00 mg), iron (0.05 mg), magnesium (13.00 mg) and sodium (49.00 mg) [71]. Due to the FAO [71] document not showing the dietary fiber value of this whole cow’s milk, this nutrient’s reference was established as 0.00 g/100 mL according to other milk present in the USDA FoodData Central [72].

#### 4.2.1. Energy

The energy value comes from macronutrients (carbohydrate, protein, and lipid) [23]. Considering the energy value of the cow’s milk mentioned above converted to the standard serving size of this study (66 Kcal/100 mL), most plant-based beverages (86.07%, *n* = 105) are less caloric than the cow’s milk using the same portion. Only one almond-based beverage [36], the *baru* almond-based beverage [40], two cashew nut-based beverages [23,33], three coconut-based beverages [23,54], the groundnut-based beverage [49], one rice-based beverage [57], the safflower-based beverage [49], two *sapucaia* nut-based beverages [47], both sesame seed-based beverages [34], two soy-based beverages [44,54], and the spelt-based beverage [54], presented a total energetic value ranging from 66 to 183 Kcal/100 mL equal or higher than the cow’s milk.

#### 4.2.2. Carbohydrate

Almost 27.87% (*n* = 34) of the studied beverages were higher in carbohydrate content than the cow’s milk (4.78 g/100 mL). Regarding differences in composition, the carbohydrate in cow’s milk is mostly lactose—contributes to the use of vitamin D and the absorption of calcium, phosphorus and magnesium in the intestine—while plant-based beverages are lactose-free [4,21,71] and flavor lactose substitutes most used are sugar, maltodextrin, apple juice, and agave syrup.

Regarding glycemic index (GI), Jeske et al. [66] evaluated the GI of bovine milk and commercial milk substitutes produced with almonds (*Prunus dulcis*), cashews (*Anacardium occidentale*), coconut (*Cocos nucifera*), hazelnut (*Corylus avellana*), hemp (*Cannabis sativa*), macadamia (*Macadamia* spp.), oat (*Avena sativa*), quinoa (*Chenopodium quinoa*), rice (*Oryza* spp.), and soy (*Glycine max*). The plant-based beverages GI ranged from 47.53 (Organic soya drink from Provamel) to 99.96 (Organic brown rice drink from Rude Health), while cow’s milk (Clona Dairy Product Ltd., Clonakilty, Ireland) had GI equal to 46.93 [66]. Among the beverages analyzed by Jeske et al. [66], eight based on almond (Alpro), carob/almond, cashew, macadamia, quinoa and soy (Provamel, Sojade and Alpro—whole-bean) presented low GI as well as bovine milk, and six other based on almonds, hazelnut, hemp, oat, and soy had medium GI. The analyzed beverages based on coconut and rice, which contained mostly glucose, had a high GI [66].

The GI value is usually influenced by the type of sugar, with each type having a GI value [66]. Jeske et al. [66] presented the GI of maltose (105), sucrose (61), fructose (19), and lactose (46). Some of the plant-based beverage samples mentioned above had added sugar or sweeteners, such as agave syrup (present in cashew, macadamia and quinoa samples), apple concentrate (present in the soy sample from Provamel), sucrose (present in hazelnut, almond and soy original from Alpro) and maple syrup (present in the carob/almond sample) [66]. Agave syrup and apple concentrate are high in fructose, while sucrose and maple syrup contributed to high sucrose values [66]. Additionally, products that have ingredients rich in starch are high in glucose and/or maltose. Rice-based samples presented high maltose and glucose content and sample based on oat, which contains β-glucan capable of reducing GI, was high in maltose [66].

#### 4.2.3. Protein

Of the plant-based beverages studied, 16.39% (*n* = 20) have a higher amount of protein than the cow’s milk (3.37 g/100 mL). Regarding protein quality, the Digestible Indispensable Amino Acid Score (DIAAS) is the recommended measure for analyzing it [73]. From the DIAAS cut-off values, the food can be characterized as “excellent/high” (value equal to or greater than 100) and “good/source” (between 75 and 99) [73].

The DIAAS method is indicated to replace the Protein Digestibility Corrected Amino Acid Score (PDCAAS) based on some considerations, such as using a single value of fecal crude protein digestibility in the PDCAAS method for correction for digestibility, while the DIAAS use “true ileal amino acid digestibility for the dietary indispensable amino acids” [73]. It is also pointed out an overvaluation of the protein quality of foods that contain antinutritional factors and an inadequate estimate of the protein quality of high-quality proteins due to a non-attribution of extra nutritional value by the PDCAAS method [73].

Chalupa-Krebzdak et al. [23], Scholz-Ahrens et al. [54], and Sousa and Kopf-Bolanz [65] showed DIAAS values for milk proteins and some plant-proteins (soy (*Glycine max*), rice (*Oryza* spp.), oats (*Avena satina*) and almond (*Prunus dulcis*)). In these studies, cow’s milk has a higher DIAAS value than the plant-based proteins. Among the plant-proteins, soy presented DIAAS values (84.00–90.60) closest to cow’s milk [23,54,65]. The DIAAS values of the other proteins presented were oats (54.00) [54,65], almond (40.00) [54] and rice (37.00–59.00) [23,54,65], not considered good nor excellent [73] as soy and cow’s milk.

Proteins provide amino acids that perform essential functions in the body, such as structural, defense, transport, and regulatory [23,74]. The deficiency of essential amino acids, which must be ingested in the diet, can reduce protein synthesis and physiological and biochemical changes [74]. Thus, attention is needed when replacing milk with a plant-based beverage regarding protein, and a diet needs to be planned to guarantee an adequate aminogram.

#### 4.2.4. Lipid

The percentage of plant-based beverages studied in which lipid content is higher than cow’s milk (3.76 g/100 mL) is 13.11% (*n* = 16). The lipid in cow’s milk is mainly composed of saturated fatty acids, while plant-based beverages tend to have a higher content of unsaturated fatty acids and are cholesterol-free [4,6,20,23,53]. However, there may be exceptions, as mentioned by Chalupa-Krebzdak et al. [23], Craig and Fresán [20] and Vanga and Raghavan [6] the case of coconut-based beverage that has a high content of saturated fatty acids.

The high consumption of foods rich in saturated fatty acids and cholesterol is associated with an increased risk of developing cardiovascular diseases, while the consumption of polyunsaturated fatty acids corresponds to a risk reduction factor for these diseases [74]. Despite that, studies have shown that the consumption of dairy products does not negatively affect human cardiovascular health [23,53,75]. Chalupa-Krebzdak et al. [23] mentioned as possible cause that “many nutrients in dairy products that may offset the effects of potentially harmful levels of dietary saturated fatty acids”.

The isocaloric replacement of saturated fatty acids for polyunsaturated has collaborated to reduce LDL and total cholesterol in humans [23,74]. In this sense, the lipid composition of the plant-based beverages does not have to be the same as that of cow’s milk regarding the nutritional aspect [23] but is necessary to evaluate its need considering sensory and technological aspects. Chalupa-Krebzdak et al. [23] pointed out that replacing milk with plant-based beverages, except for some coconut-based beverages, can decrease the intake of saturated fatty acids in the diet.

Although coconut-based beverage has a high content of saturated fatty acids [6,20,23], it contains medium-chain fatty acids (MCFAs) that can be converted into ketone compounds, which are favorable in brain functioning [14]. In addition, Vanga and Raghavan [6] report that lauric acid, which mainly contributes to raise high-density lipoprotein (HDL) cholesterol levels and, consequently, reduce levels in the bloodstream of low-density lipoprotein (LDL) cholesterol, is present in coconut fats.

#### 4.2.5. Dietary Fiber

The consumption of dietary fiber might contribute to intestinal regulation, the reduction of blood cholesterol and glucose, and it is also associated with a lower incidence of diabetes, cardiovascular disease, gastrointestinal disorders and colon cancer [74,76]. Among the plant-based beverages that present dietary fiber data, most (81.82%, *n* = 18) have a higher dietary fiber content than cow’s milk reference (0.00 g/100 mL). In this sense, plant-based beverages have an advantage. Unfortunately, due to the studies’ lack of data, it was not possible to link the fiber content and GI in the samples analyzed.

#### 4.2.6. Micronutrients

Calcium is a nutrient that stands out in milk and is essential for bone and dental structure, muscle function, and nerve conduction [54,74]. The calcium values of plant-based beverages ranged from 0.00 to 1252.94 mg/100 mL and 44.44% (*n* = 24) of the plant-based beverages were greater than the cow’s milk (122.21 mg/100 mL). These beverages with a higher calcium content (except for the beverages based on *sapucaia* nut, which went through crioconcentration as previously mentioned), were all fortified with calcium.

As mentioned earlier about fortification, many commercial plant-based beverages are fortified with calcium to achieve cow’s milk’s amount [21]. Despite this, there are plant-based beverages that are not fortified. The substitution of milk for these non-fortified beverages when the diet is not balanced, calcium and other nutrients may be deficient [21].

Moreover, the bioavailability of the fortifier, and not only the amount of the nutrient, should also be taken into account [54]. As an example of calcium, Craig and Fresán [20] mention that calcium absorption from tricalcium phosphate is considerably less than milk, while calcium carbonate is equivalent to milk.

Magnesium is another essential micronutrient contained in milk [4]. Considering the studied beverages in which the magnesium data were provided (ranging from 0.84 to 10,178.60 mg/100 mL), most of them (87.50%, *n* = 14) have higher magnesium content than cow’s milk (13.35 mg/100 mL). The *sapucaia* nut-based beverages have a very high magnesium content per 100 mL, exceeding the Tolerable Upper Intake Level (UL) for this nutrient [77]. Therefore, as previously mentioned, these beverages can be used as ingredients in other food products for nutritional purposes [47].

Iron is not naturally contained in appreciable amounts in cow’s milk [71]. Considering iron content of the beverages that present these data (ranging from 0.04 to 1.40 mg/100 mL), only a rice-based beverage (12.50%, *n* = 1) [46] presented lower amount, however close, than the whole cow’s milk (0.05 mg/100 mL). With that, plant-based beverages have a certain advantage in terms of quantity, mainly considering a vegetarian diet that, in general, lacks iron [78].

Sodium is a component of salt and it is also found in milk [28]. Craig and Fresán [20] pointed out that consumers are generally concerned about the content of this mineral in plant-based beverages for health reasons. The high consumption of this nutrient is associated with noncommunicable diseases [28]. Among the plant-based beverages that present sodium data that ranged from 0.00 to 343.43 mg/100 mL, 53.57% (*n* = 15) of the beverages have higher sodium values than cow’s milk (50.32 mg/100 mL). These beverages with a higher sodium content (except for the beverages based on *sapucaia* nut, which went through the process of crioconcentration as previously mentioned [47]), are commercial beverages that have salt as an ingredient [52]. Despite this, other beverages contain salt as an ingredient that has a lower sodium content than this cow’s milk [52,55].

Unfortunately, the function of salt in these beverages that present sodium information and have salt as an ingredient has not been reported. However, Karimidastjerd and Kilic-Akyilmaz [60] reported adding salt to the beverage to balance the flavor, and Pinelli et al. [79] used salt to produce a quinoa-based beverage to increase the protein content.

### 4.3. Limitations

Lastly, the main limitation of this study is that not all studies provided the information in a more homogeneous way, which did not allow the realization of more elaborate statistics and the grouping of data. Additionally, as limitation, the lack of some data on the micronutrients studied and more information on the quality/type of nutrients for all types of beverages studied for a complete analysis.

## 5. Conclusions

The demand for plant-based beverages used as substitutes for cow’s milk is increasing worldwide. In that sense, there is a wide variety of these beverages and new ones are constantly emerging. Despite some similarities to cow’s milk, such as appearance, this review showed that the nutritional aspects of these beverages vary widely (energy value varied from 6 to 183 Kcal/100 mL; carbohydrate from 0.00 to 22.29 g/100 mL; protein from 0.06 to 12.43 g/100 mL; lipid from 0.00 to 19.00 g/100 mL; dietary fiber from 0.00 to 4.40 g/100 mL; calcium from 0.00 to 1252.94 mg/100 mL; iron from 0.04 to 1.40 mg/100 mL; magnesium from 0.84 to 10,178.60 mg/100 mL; sodium from 0.00 to 343.43 mg/100 mL).

Our initial hypothesis, that plant-based beverages are lower in protein and calcium than cow’s milk, was partially confirmed given that some samples presented similar or higher protein and calcium content than cow’s milk. A commercial soy-beverage (with apple concentrate, algae *Lithothamnium calcareum*, and salt) and *sapucaia* nut-based beverage (under crioconcentration process) exceeded the amount of protein and calcium compared to cow’s milk, proving to be good alternatives regarding these parameters.

Different types of raw material used, the added ingredients, the extraction process, and the treatments were used to improve the nutritional content of plant-based beverages aiming to be healthier and similar to cow’s milk. Soy is the most common matrix used in the plant-based beverages studied. Based on the ingredient list of the studied products, salt was the added ingredient that most frequently appeared, followed by sugar. However, considering all the sources of ingredients used to sweeten plant-based beverages, all of them presented a type (or combination) of sweetener ingredient, probably in higher amounts than salt aiming to improve sensory characteristics and acceptance. However, it was not possible to analyze it in this study due to the lack of information in some studies about the quantity of the ingredients. Further studies are necessary to evaluate the amount of each ingredient in plant-based beverages and its impacts on individuals’ health.

It is necessary to pay attention to the substitution of cow’s milk by these alternatives considering the nutritional quality. Due to the diversity of nutrients’ type and amount of nutrients found in the studies analyzed, it is noteworthy that most plant-based beverages cannot completely fulfill the replacement of cow’s milk regarding nutritional quality. Some of them present ingredients (legumes, almonds, nuts, seeds, etc.) that can also be allergenic for some individuals and their use should be evaluated with caution for allergic individuals. Therefore, having nutritional monitoring is important for an adequate replacement of cow’s milk in the diet. The choice of plant-based beverage will depend on the objective (nutritional or sensory) that the person is looking for to use this product and their preferences/restrictions. Thereby, this study can be useful in choosing the best alternative to compose their diet.

## Figures and Tables

**Table 1:** Table of nutritional composition (per 100 mL), ingredients and specifications on the origin of plant-based beverages from scientific studies selected according to the inclusion and exclusion criteria.

Authors and Year	Ingredients	Energy (Kcal)	CHO (g)	Protein (g)	Lipid (g)	Dietary Fiber (g)	Ca (mg)	Fe (mg)	Mg (mg)	Na (mg)	Specifications on the Origin of the Beverage/Where the Nutritional Data Were Obtained
**Almond-based beverage**											
Alozie and Udofia, 2015	Water, almond (dehulled), sugar syrup (granulated sugar + water), vanilla essence.	55	4.50	1.70	3.40	1.25	13.10	1.40	42.05	6.38	Beverage prepared and analyzed for the study.
Chalupa-Krebzdak et al., 2018	Almond milk (filtered water, almonds), evaporated cane juice syrup, calcium carbonate, sea salt, potassium citrate, carrageenan, sunflower lecithin, vitamin A palmitate, vitamin D2, D-alpha tocopherol (natural vitamin E).	25	3.33	0.42	1.04	_	188.00	_	_	_	USDA Food Composition Database.
Chalupa-Krebzdak et al., 2018	Almond milk (filtered water, almonds), calcium carbonate, tapioca starch, sea salt, potassium citrate, carrageenan, sunflower lecithin, natural flavour, vitamin A palmitate, vitamin D2 and D-alpha tocopherol (natural vitamin E).	12	0.62	0.31	1.08	_	185.00	_	_	_	USDA Food Composition Database.
Chalupa-Krebzdak et al., 2018	Almond milk (filtered water, almonds), honey, cane sugar, calcium carbonate, sea salt, potassium citrate, carrageenan, sunflower lecithin, guar gum, natural flavor, vitamin A palmitate, vitamin D2, D-alpha tocopherol (natural vitamin E).	21	3.33	0.42	1.04	_	188.00	_	_	_	USDA Food Composition Database.
Chalupa-Krebzdak et al., 2018	Almond milk (water, almonds), pea protein, rice protein, calcium phosphate, magnesium phosphate, carrageenan, natural flavor, locust bean gum, kosher sea salt, vitamin A palmitate, vitamin D2 L-selenomethionine (selenium), zinc oxide, folic acid, vitamin b-12.	17	0.42	2.08	0.83	_	42.00	_	_	_	USDA Food Composition Database.
Decloedt et al., 2018	Water, almond (2.10%), tricalcium phosphate, salt, sunflower lecithine (emulsifier), sugar, locust bean gum, gellan gum.	24	3.00	0.50	1.10	0.20	120.00	_	_	56.00 ^a^	Nutritional values obtained from the label of the drinks that were purchased.
Decloedt et al., 2018	Water, almond (2.10%), tricalcium phosphate, salt, sunflower lecithine (emulsifier), aromas, locust bean gum, gellan gum.	13	0.10	0.50	1.30	0.20	120.00	_	_	56.00 ^a^	Nutritional values obtained from the label of the drinks that were purchased.
Jeske, 2018	Water, sugar, almond (2.00%), tri-calcium phosphate, sea salt, stabilizers (locust bean gum, gellan gum), emulsifier (sunflower lecithin), vitamins B2, B12, E, D2).	25 ^b^	3.08 ^b^	0.51 ^b^	1.13 ^b^	_	_	_	_	_	Nutritional values obtained from the label. Brand name: Alpro.
Jeske, 2018	Water, almond (7.00%), sea salt.	34 ^b^	0.21 ^b^	0.92 ^b^	3.08 ^b^	_	_	_	_	_	Nutritional values obtained from the label. Brand name: Provamel.
Martínez-Padilla et al., 2020	Water, almond (7.00%), tapioca starch, natural almond flavoring.	32	3.30	1.00	2.10	_	_	_	_	_	Nutritional values obtained from the label. Brand name: Ecomil.
Scholz-Ahrens et al., 2020	Water, almonds (6.50%) sea salt.	32 ^b^	0.21 ^b^	0.82 ^b^	2.98 ^b^	_	ND	_	_	_	Nutritional values obtained from the label. Brand name: Provamel.
Silva, 2018	Water, almonds.	68 ^b^	0.67 ^b^	4.36 ^b^	5.51 ^b^	2.16 ^b^	_	_	_	_	Beverage prepared and analyzed for the study.
**Amaranth-based beverage**											
Manassero et al., 2020	Water, amaranth seeds, xanthan gum, gellan gum.	32 ^b^	3.15 ^b^	3.51 ^b^	0.62 ^b^	1.95 ^b^	14.87 ^b^	0.76 ^b^	_	_	Beverage prepared and analyzed for the study.
**Baru almond-based beverage**											
Vieira, 2017	Water, baru almond.	71 ^b^	1.94 ^b^	3.15 ^b^	5.66 ^b^	_	_	_	_	_	Beverage prepared and analyzed for the study.
**Cashew nut-based beverage**											
Chalupa-Krebzdak et al., 2018	Cashew milk (filtered water, cashews), cane sugar, tricalcium phosphate, sea salt, almond butter, locust bean gum, sunflower lecithin, gellan gum, vitamin E acetate, zinc gluconate, vitamin A palmitate, riboflavin (B2) vitamin B12, vitamin D2.	25	3.75	0.42	1.04	_	188.00	_	_	_	USDA Food Composition Database.
Chalupa-Krebzdak et al., 2018	Cashew milk (filtered water, cashews), cane sugar, tricalcium phosphate, sea salt, almond butter, locus bean gum, sunflower lecithin, gellan gum, vitamin E acetate, zinc gluconate, vitamin A palmitate, riboflavin (B2) vitamin B12, vitamin D2.	79	5.73	2.20	5.29	_	9.00	_	_	_	USDA Food Composition Database.
Decloedt et al., 2018	Water, cashew nuts (3.10%), tricalcium phosphate, salt, sunflower lecithine (emulsifier), sugar, locust bean gum, gellan gum.	23	2.60	0.50	1.10	0.20	120.00	_	_	52.00 ^a^	Nutritional values obtained from the label of the drinks that were purchased.
Holanda, 2017	Water, cashew nut, sugar, tricalcium calcium phosphate.	58 ^b^	4.92 ^b^	2.13 ^b^	3.29 ^b^	_	108.76 ^b^	_	_	_	Beverage prepared and analyzed for the study.
Jeske, 2018	Water, roasted cashew (6.00%), agave syrup (3.50%), sea salt.	48 ^b^	4.52 ^b^	0.92 ^b^	2.88 ^b^	_	_	_	_	_	Nutritional values obtained from the label. Brand name: Provamel.
Lima et al., 2020	Water, broken cashew nut kernels, sugar.	66 ^b^	5.58 ^b^	1.88 ^b^	4.08 ^b^	_	_	_	_	_	Beverage prepared and analyzed for the study.
Manzoor et al., 2017	Water, cashew nuts, sugar syrup, vanilla flavor.	57 ^b^	4.50 ^b^	2.11 ^b^	3.39 ^b^	1.18 ^b^	21.90	0.80	38.20	22.80	Beverage prepared and analyzed for the study.
Scholz-Ahrens et al., 2020	Water, cashew nuts (3.10%), sugar, calcium (tri-calcium phosphate), sea salt, stabilizers (locust bean gum, gellan gum), emulsifier (sunflower lecithin), vitamins (riboflavin (B2), B12, E, D2).	24 ^b^	2.67 ^b^	0.51 ^b^	1.13 ^b^	_	ND	_	_	_	Nutritional values obtained from the label. Brand name: Alpro.
**Coconut-based beverage**											
Chalupa-Krebzdak et al., 2018	Organic coconut milk (or water, organic coconut cream), organic dried cane syrup, chicory root extract (inulin), tapioca dextrose, pectin, algin (kelp extract), magnesium phosphate, tricalcium phosphate, rice starch, natural flavours, locust bean gum, live cultures, carrageenan, guar gum, dipotassium phosphate, vitamin B12.	76	9.41	0.59	4.12	_	176.00	_	_	_	USDA Food Composition Database.
Chalupa-Krebzdak et al., 2018	Coconut extract (25.00%), water, carboxymethyl cellulose (E466, guar gum.	92	7.00	2.00	6.00	_	0.00	_	_	_	USDA Food Composition Database.
Chalupa-Krebzdak et al., 2018	Coconut milk, water, stabilizer, sodium metabisulphite.	50	3.75	1.25	5.00	_	0.00	_	_	_	USDA Food Composition Database.
Decloedt et al., 2018	Water, coconut milk (coconut cream and water) (5.30%), rice (3.30%), tricalcium phosphate, salt (sea), aromas, carrageenan, guar gum, xanthan gum.	20	2.70	0.10	0.90	0.00	120.00	_	_	52.00 ^a^	Nutritional values obtained from the label of the drinks that were purchased.
Jeske, 2018	Water, coconut milk (5.30%) (coconut cream, water), rice (3.30%), tri-calcium phosphate, stabilizers (carrageenan, guar gum, Xanthan gum), sea salt, vitamins (B12, D2), flavorings.	21 ^b^	2.77 ^b^	0.10 ^b^	0.92 ^b^	_	_	_	_	_	Nutritional values obtained from the label. Brand name: Alpro.
Martínez-Padilla et al., 2020	Water, coconut milk (5.30%), raw cane sugar, maltodextrin, algae *Lithothamnium calcareum*.	26	4.10	0.10	0.90	_	_	_	_	_	Nutritional values obtained from the label. Brand name: Naturli.
Scholz-Ahrens et al., 2020	Coconut extract 85.00%, water.	183 ^b^	2.05 ^b^	1.64 ^b^	19.00 ^b^	_	ND	_	_	_	Nutritional values obtained from the label. Brand name: Real Thai.
Scholz-Ahrens et al., 2020	Coconut milk (30.00%), water, corn starch.	46 ^b^	0.51 ^b^	0.51 ^b^	4.62 ^b^	_	ND	_	_	_	Nutritional values obtained from the label. Brand name: Renuka.
**Cupuaçu almond-based beverage**											
*Silva* et al., 2015	Water, cupuaçu almond flour.	7 ^b^	0.26 ^b^	0.10 ^b^	0.63 ^b^	_	_	_	_	_	Beverage prepared (extraction temperature of 55 °C) and analyzed for the study.
*Silva* et al., 2015	Water, cupuaçu almond flour.	9 ^b^	0.46 ^b^	0.07 ^b^	0.81 ^b^	_	_	_	_	_	Beverage prepared (extraction temperature of 75 °C) and analyzed for the study.
*Silva* et al., 2015	Water, cupuaçu almond flour.	10 ^b^	0.77 ^b^	0.13 ^b^	0.75 ^b^	_	_	_	_	_	Beverage prepared (extraction temperature of 100 °C) and analyzed for the study.
**Groundnut-based beverage**											
Meeshi et al., 2014	Water, groundnut, sodium bicarbonate (1.00% solution).	72	4.13	3.10	4.80	_	33.47	_	_	_	Beverage prepared and analyzed for the study.
**Hazelnut-based beverage**											
Jeske, 2018	Water, sugar, hazelnuts (2.50%), tri-calcium phosphate, sea salt, stabilizers (locust bean gum, gellan gum), emulsifier (sunflower lecithin), vitamins B2, B12, E, D2).	30 ^b^	3.18 ^b^	0.41 ^b^	1.64 ^b^	_	_	_	_	_	Nutritional values obtained from the label. Brand name: Alpro.
Martínez-Padilla et al., 2020	Water, sugar, hazelnuts (2.50%), calcium (tri-calcium phosphate), sea salt, stabilizers (locust bean gum, gellan gum), emulsifier (sunflower lecithin), vitamins (riboflavin B2, B12, E, D2).	29	3.10	0.40	1.60	_	_	_	_	_	Nutritional values obtained from the label. Brand name: Alpro.
Scholz-Ahrens et al., 2020	Water, sugar, hazelnuts (2.50%), calcium (tri-calcium phosphate), sea salt, stabilizers (locust bean gum, gellan gum), emulsifier (sunflower lecithin), vitamins (riboflavin (B2), B12, E, D2).	30 ^b^	3.18 ^b^	0.41 ^b^	1.64 ^b^	_	123.24 ^b^	_	_	_	Nutritional values obtained from the label. Brand name: Alpro.
Scholz-Ahrens et al., 2020	Water, European hazelnuts (4.00%), agave syrup (3.50%), sea salt.	37 ^b^	2.67 ^b^	0.62 ^b^	2.67 ^b^	_	ND	_	_	_	Nutritional values obtained from the label. Brand name: Provamel.
**Hemp-based beverage**											
Chalupa-Krebzdak et al., 2018	Organic flax/hemp cream (filtered water, organic flax oil, organic hemp oil), organic brown rice solids, organic brown rice syrup, organic tapioca, non-GMO sunflower lecithin, Himalayan salt, organic guar gum, xanthan gum.	19	2.50	0.83	1.25	_	12.00	_	_	_	USDA Food Composition Database.
Jeske, 2018	Water, hemp cream (3.00%), tri-calcium phosphate, emulsifier (sucrose ester), natural flavoring, stabilizer (xanthan gum), sea salt, stabilizer (gellan gum), vitamin D2.	24 ^b^	0.10 ^b^	0.10 ^b^	2.77 ^b^	_	_	_	_	_	Nutritional values obtained from the label. Brand name: Braham and Murray.
Martínez-Padilla et al., 2020	Water, hemp seeds (3.00%), hemp oil (1.30%), tapioca starch, emulsifier: sunflower lecithin.	40	2.20	1.00	2.90	_	_	_	_	_	Nutritional values obtained from the label. Brand name: Ecomil.
**Macadamia nut-based beverage**											
Jeske, 2018	Water, macadamia nuts (4.00%), agave syrup (3.50%), sea salt.	35 ^b^	2.46 ^b^	0.51 ^b^	2.46 ^b^	_	_	_	_	_	Nutritional values obtained from the label. Brand name: Provamel.
Scholz-Ahrens et al., 2020	Water, macadamia nuts (4.00%), agave syrup (3.50%), sea salt.	38 ^b^	2.77 ^b^	0.31 ^b^	2.67 ^b^	_	ND	_	_	_	Nutritional values obtained from the label. Brand name: Provamel.
**Millet-based beverage**											
Scholz-Ahrens et al., 2020	Water, millet (12.00%), high-oleic sunflower oil, sea salt.	51 ^b^	9.24 ^b^	0.51 ^b^	1.54 ^b^	_	ND	_	_	_	Nutritional values obtained from the label. Brand name: Swiss cereal drink.
**Oat-based beverage**											
Andrade, 2018	Water, oats.	35 ^b^	1.00 ^b^	1.86 ^b^	2.69 ^b^	3.21 ^b^	_	_	_	_	Beverage prepared and analyzed for the study.
Decloedt et al., 2018	Water, oats (16.00%), sunflower oil, tricalcium phosphate, salt (sea), acacia gum (gum arabic).	52	8.90	0.40	1.40	1.00	120.00	_	_	48.00 ^a^	Nutritional values obtained from the label of the drinks that were purchased.
Decloedt et al., 2018	Water, oats (10.00%), canola oil, calcium carbonate, tricalcium phosphate (and other), salt.	45	6.50	1.00	1.50	0.80	120.00	_	_	40.00 ^a^	Nutritional values obtained from the label of the drinks that were purchased.
Jeske, 2018	Oat base (water, oats 10.00%), sea salt.	36 ^b^	6.68 ^b^	1.03 ^b^	0.51 ^b^	_	_	_	_	_	Nutritional values obtained from the label. Brand name: Oatly.
Martínez-Padilla et al., 2020	Water, oats (10.00%), sea salt.	36	6.50	1.00	0.50	_	_	_	_	_	Nutritional values obtained from the label. Brand name: Oatly organic.
Ravindran and RadhaiSri, 2020	Water, oats.	33	7.30	0.89	0.37	4.40	_	_	_	_	Beverage prepared and analyzed for the study.
Scholz-Ahrens et al., 2020	Water, oat (10.00%), rapeseed oil, algae (*Lithothamnium calcareum*), sea salt, citric acid.	46 ^b^	6.68 ^b^	1.03 ^b^	1.54 ^b^	_	123.24 ^b^	_	_	_	Nutritional values obtained from the label. Brand name: Oatly.
**Quinoa-based beverage**											
Jeske, 2018	Water, quinoa (7.00%), agave syrup, corn maltodextrin, almond oil.	47 ^b^	3.80 ^b^	1.54 ^b^	2.88 ^b^	_	_	_	_	_	Nutritional values obtained from the label. Brand name: EcoMil.
Martínez-Padilla et al., 2020	Water, quinoa (4.00%), inulin (agave fiber), sunflower oil, emulsifier: sunflower lecithin.	29	3.50	0.50	1.20	_	_	_	_	_	Nutritional values obtained from the label. Brand name: Ecomil.
Vieira, 2013	Water (distilled), quinoa, saline solution 0.03 M (sodium chloride + distilled water), Termamyl enzyme, amyloglucosidase enzyme, sunflower oil (1.00%).	35	5.47	1.02	1.04	_	_	_	_	_	Beverage prepared and analyzed for the study.
**Rice-based beverage**											
Abrão, 2019	Water, brown rice.	20 ^b^	4.20 ^b^	0.40 ^b^	0.00 ^b^	0.35 ^b^	0.00 ^b^	_	_	0.00 ^b^	Beverage prepared for the study. Nutritional values based on the label of the product of origin.
Abrão, 2019	Water, parboiled rice.	21 ^b^	4.60 ^b^	0.40 ^b^	0.00 ^b^	0.00 ^b^	0.00 ^b^	_	_	0.65 ^b^	Beverage prepared for the study. Nutritional values based on the label of the product of origin.
Abrão, 2019	Water, polished rice.	21 ^b^	5.00 ^b^	0.35 ^b^	0.00 ^b^	0.00 ^b^	2.50 ^b^	_	_	0.00 ^b^	Beverage prepared for the study. Nutritional values based on the label of the product of origin.
Abrão, 2019	Water, red rice.	21 ^b^	4.20 ^b^	0.35 ^b^	0.00 ^b^	0.00 ^b^	0.00 ^b^	_	_	0.00 ^b^	Beverage prepared for the study. Nutritional values based on the label of the product of origin.
Carvalho et al., 2011	Water, broken polished rice of the EPAGRI 109 variety.	18 ^b^	3.25 ^b^	0.75 ^b^	0.42 ^b^	_	0.90 ^b^	0.04 ^b^	0.84 ^b^	_	Beverage prepared and analyzed for the study.
Carvalho et al., 2011	Water, brown rice.	21 ^b^	3.13 ^b^	0.86 ^b^	0.61 ^b^	_	1.24 ^b^	0.08 ^b^	1.73 ^b^	_	Beverage prepared and analyzed for the study.
Decloedt et al., 2018	Water, rice (13.40%), sunflower oil, calcium carbonate, salt, gellan gum.	58	12.00	0.20	1.00	0.30	120.00	_	_	40.00 ^a^	Nutritional values obtained from the label of the drinks that were purchased.
Jeske, 2018	Organic rice, water, organic sunflower oil, sea salt.	66 ^b^	10.78 ^b^	0.72 ^b^	1.95 ^b^	_	_	_	_	_	Nutritional values obtained from the label. Brand name: Vitariz.
Jeske, 2018	Water, organic brown rice (14.00%), sunflower oil, sea salt.	61 ^b^	11.30 ^b^	0.31 ^b^	1.34 ^b^	_	_	_	_	_	Nutritional values obtained from the label. Brand name: Rude Health.
Karimidastjerd and Kilic-Akyilmaz, 2021	Water (distilled), white rice flour (3.00–8.00%, *w*/*w*), xanthan gum (0.01–0.05%, *w*/*w*), sunflower oil (1.00%, *w*/*w*), sea salt (0.10%, *w*/*w*).	25 ^b^	2.57 ^b^	0.21 ^b^	1.54 ^b^	_	_	_	_	_	Beverage prepared and analyzed for the study.
Karimidastjerd and Kilic-Akyilmaz, 2021	Water (distilled), white rice flour (3.00–8.00%, *w*/*w*), xanthan gum (0.01–0.05%, *w*/*w*), sunflower oil (1.00%, *w*/*w*), sea salt (0.10%, *w*/*w*), sugar (2.50%, *w*/*v*).	35 ^b^	5.14 ^b^	0.21 ^b^	1.54 ^b^	_	_	_	_	_	Beverage prepared and analyzed for the study.
Martínez-Padilla et al., 2020	Water, rice (11.00%), sunflower oil, sea salt.	54	11.00	0.10	1.10	_	_	_	_	_	Nutritional values obtained from the label. Brand name: Naturli.
Scholz-Ahrens et al., 2020	Water, European rice (17.00%), coconut milk (4.00%), sea salt.	62 ^b^	12.84 ^b^	0.31 ^b^	0.92 ^b^	_	ND	_	_	_	Nutritional values obtained from the label. Brand name: Provamel.
Storck and Montagner, 2020	Water, broken polished rice, vanilla essence, salt.	40 ^b^	9.01 ^b^	0.97 ^b^	0.04 ^b^	1.09 ^b^	_	_	_	_	Beverage prepared and analyzed for the study.
**Safflower-based beverage**											
Meeshi et al., 2014	Water, safflower seed, sodium hexameta phosphate (0.20%), salt.	70	2.62	2.40	5.62	_	55.30	_	_	_	Beverage prepared and analyzed for the study.
**Sapucaia nut-based beverage**											
Demoliner, 2019	Water (distilled), sapucaia nut pie.	54 ^b^	1.34 ^b^	1.94 ^b^	4.51 ^b^	_	636.74 ^b^	_	2916.68 ^b^	315.80 ^b^	Beverage prepared (submitted to the crioconcentration method—initial volume) and analyzed for the study.
Demoliner, 2019	Water (distilled), sapucaia nut pie.	40 ^b^	2.10 ^b^	2.36 ^b^	2.42 ^b^	_	709.66 ^b^	_	3183.70 ^b^	318.78 ^b^	Beverage prepared (submitted to the crioconcentration method—concentrated fluid 1) and analyzed for the study.
Demoliner, 2019	Water (distilled), sapucaia nut pie.	60 ^b^	1.03 ^b^	2.19 ^b^	5.26 ^b^	_	523.77 ^b^	_	1008.51 ^b^	120.06 ^b^	Beverage prepared (submitted to the crioconcentration method—ice 1) and analyzed for the study.
Demoliner, 2019	Water (distilled), sapucaia nut pie.	50 ^b^	3.97 ^b^	2.67 ^b^	2.55 ^b^	_	738.41 ^b^	_	3382.94 ^b^	325.66 ^b^	Beverage prepared (submitted to the crioconcentration method—concentrated fluid 2) and analyzed for the study.
Demoliner, 2019	Water (distilled), sapucaia nut pie.	45 ^b^	2.67 ^b^	0.91 ^b^	3.43 ^b^	_	517.61 ^b^	_	1283.75 ^b^	192.25 ^b^	Beverage prepared (submitted to the crioconcentration method—ice 2) and analyzed for the study.
Demoliner, 2019	Water (distilled), sapucaia nut pie.	56 ^b^	4.29 ^b^	4.40 ^b^	2.32 ^b^	_	862.99 ^b^	_	4467.45 ^b^	323.71 ^b^	Beverage prepared (submitted to the crioconcentration method—concentrated fluid 3) and analyzed for the study.
Demoliner, 2019	Water (distilled), sapucaia nut pie.	21 ^b^	2.75 ^b^	0.84 ^b^	0.69 ^b^	_	515.55 ^b^	_	1345.37 ^b^	262.09 ^b^	Beverage prepared (submitted to the crioconcentration method—ice 3) and analyzed for the study.
Demoliner, 2019	Water (distilled), sapucaia nut pie.	76 ^b^	3.19 ^b^	7.57 ^b^	3.65 ^b^	_	952.23 ^b^	_	6264.70 ^b^	331.82 ^b^	Beverage prepared (submitted to the crioconcentration method—concentrated fluid 4) and analyzed for the study.
Demoliner, 2019	Water (distilled), sapucaia nut pie.	11 ^b^	1.01 ^b^	0.77 ^b^	0.46 ^b^	_	313.24 ^b^	_	945.87 ^b^	163.50 ^b^	Beverage prepared (submitted to the crioconcentration method—ice 4) and analyzed for the study.
Demoliner, 2019	Water (distilled), sapucaia nut pie.	119 ^b^	7.98 ^b^	12.43 ^b^	4.12 ^b^	_	1252.94 ^b^	_	10,178.60 ^b^	343.43 ^b^	Beverage prepared (submitted to the crioconcentration method—concentrated fluid 5) and analyzed for the study.
Demoliner, 2019	Water (distilled), sapucaia nut pie.	22 ^b^	1.83 ^b^	2.00 ^b^	0.77 ^b^	_	752.79 ^b^	_	1663.74 ^b^	292.90 ^b^	Beverage prepared (submitted to the crioconcentration method—ice 5) and analyzed for the study.
**Sesame seed-based beverage**											
Reis, 2019	Water (distilled), sesame seeds.	139 ^b^	12.77 ^b^	5.55 ^b^	7.26 ^b^	_	_	_	_	_	Beverage prepared and analyzed for the study.
Reis, 2019	Water (distilled), sesame seeds, maltodextrin (10.00%).	170 ^b^	22.29 ^b^	5.23 ^b^	6.69 ^b^	_	_	_	_	_	Beverage prepared and analyzed for the study.
**Soy-based beverage**											
Barros, 2012	Water, soybean (cultivar Embrapa BRS-213).	44 ^b^	1.44 ^b^	4.50 ^b^	2.31 ^b^	_	_	_	_	_	Beverage prepared (control) and analyzed for the study.
Barros, 2012	Water, soybean (cultivar Embrapa BRS-213), tocopherol.	51 ^b^	1.53 ^b^	4.83 ^b^	2.83 ^b^	_	_	_	_	_	Beverage prepared (subjected to 5.00 kGy of irradiation with tocopherol supplementation) and analyzed for the study.
Barros, 2012	Water, soybean (cultivar Embrapa BRS-213), tocopherol.	54 ^b^	1.70 ^b^	4.99 ^b^	2.99 ^b^	_	_	_	_	_	Beverage prepared (subjected to a temperature of 80 °C with tocopherol supplementation) and analyzed for the study.
Barros, 2012	Water, soybean (cultivar Embrapa BRS-258).	50 ^b^	1.77 ^b^	4.49 ^b^	2.82 ^b^	_	_	_	_	_	Beverage prepared (control) and analyzed for the study.
Barros, 2012	Water, soybean (cultivar Embrapa BRS-258), tocopherol.	51 ^b^	1.69 ^b^	4.74 ^b^	2.79 ^b^	_	_	_	_	_	Beverage prepared (subjected to 5.00 kGy of irradiation with tocopherol supplementation) and analyzed for the study.
Barros, 2012	Water, soybean (cultivar Embrapa BRS-258), tocopherol.	51 ^b^	1.67 ^b^	4.57 ^b^	2.94 ^b^	_	_	_	_	_	Beverage prepared (subjected to a temperature of 80 °C with tocopherol supplementation) and analyzed for the study.
Barros, 2012	Water, soybean (cultivar Embrapa Emb-48).	50 ^b^	1.87 ^b^	4.28 ^b^	2.88 ^b^	_	_	_	_	_	Beverage prepared (control) and analyzed for the study
Barros, 2012	Water, soybean (cultivar Embrapa Emb-48), tocopherol.	50 ^b^	1.80 ^b^	4.25 ^b^	2.82 ^b^	_	_	_	_	_	Beverage prepared (subjected to 5.00 kGy of irradiation with tocopherol supplementation) and analyzed for the study.
Barros, 2012	Water, soybean (cultivar Embrapa Emb-48), tocopherol.	54 ^b^	1.91 ^b^	4.54 ^b^	3.17 ^b^	_	_	_	_	_	Beverage prepared (subjected to a temperature of 80 °C with tocopherol supplementation) and analyzed for the study.
Barros, 2016	Water, soybean (cultivar Embrapa BRS-213), acacia/arabic gum (3.00%), neutral alloy (guar and carboxymethylcellulose) (1.00%), vanilla essence (0.20%), tocopherol, ascorbic acid, concentrated apple juice.	61 ^b^	12.38 ^b^	1.32 ^b^	0.65 ^b^	_	_	_	_	_	Beverage prepared (control) and analyzed for the study.
Barros, 2016	Water, soybean (cultivar Embrapa BRS-213), acacia/arabic gum (3.00%), neutral alloy (guar and carboxymethylcellulose) (1.00%), vanilla essence (0.20%), tocopherol, ascorbic acid, concentrated apple juice.	60 ^b^	12.20 ^b^	1.20 ^b^	0.75 ^b^	_	_	_	_	_	Beverage prepared (subjected to 2.00 kGy of gamma radiation) and analyzed for the study.
Barros, 2016	Water, soybean (cultivar Embrapa BRS-213), acacia/arabic gum (3.00%), neutral alloy (guar and carboxymethylcellulose) (1.00%), vanilla essence (0.20%), tocopherol, ascorbic acid, concentrated apple juice.	61 ^b^	12.18 ^b^	1.12 ^b^	0.82 ^b^	_	_	_	_	_	Beverage prepared (subjected to 4.00 kGy of gamma radiation) and analyzed for the study.
Barros, 2016	Water, soybean (cultivar Embrapa BRS-213), acacia/arabic gum (3.00%), neutral alloy (guar and carboxymethylcellulose) (1.00%), vanilla essence (0.20%), tocopherol, ascorbic acid, concentrated apple juice.	60 ^b^	12.41 ^b^	0.99 ^b^	0.70 ^b^	_	_	_	_	_	Beverage prepared (subjected to 8.00 kGy of gamma radiation) and analyzed for the study.
Barros and Venturini Filho, 2016	Water, soybean.	26 ^b^	0.41 ^b^	2.77 ^b^	1.44 ^b^	_	_	_	_	_	Beverage prepared (cold grinding method—aluminum cauldron) and analyzed for the study.
Barros and Venturini Filho, 2016	Water, soybean.	31 ^b^	1.54 ^b^	3.18 ^b^	1.44 ^b^	_	_	_	_	_	Beverage prepared (hot grinding method—mechanical cow) and analyzed for the study.
Barros and Venturini Filho, 2016	Water, soybean, sugar.	62 ^b^	10.27 ^b^	2.57 ^b^	1.23 ^b^	_	_	_	_	_	Beverage prepared (cold grinding method—aluminum cauldron) and analyzed for the study.
Barros and Venturini Filho, 2016	Water, soybean, sugar.	67 ^b^	10.37 ^b^	3.08 ^b^	1.34 ^b^	_	_	_	_	_	Beverage prepared (hot grinding method—mechanical cow) and analyzed for the study.
Chalupa-Krebzdak et al., 2018	Soymilk 97.20% (purified water, soy beans), coconut oil, sugar, water, salt, glycerin mono fatty acid ester, sodium bicarbonate.	58	2.63	3.16	3.68	_	0.00	_	_	_	USDA Food Composition Database.
Chalupa-Krebzdak et al., 2018	Organic soymilk (filtered water, whole organic soy beans), calcium carbonate, organic locust bean gum, sea salt, natural flavours, gellan gum, vitamin A palmitate, ergocalciferol (vitamin D2), riboflavin (vitamin B2), cyanoconalamin (vitamin B12).	33	1.67	2.92	1.67	_	125.00	_	_	_	USDA Food Composition Database.
Chalupa-Krebzdak et al., 2018	Soy milk (filtered water, soy beans), cane sugar, contains 2.00% or less of: vitamin and mineral blend (calcium carbonate, vitamin A palmitate, vitamin D2, riboflavin B2, vitamin B12), sea salt, natural flavor, gellan gum.	42	5.00	2.50	1.46	_	188.00	_	_	_	USDA Food Composition Database.
Chalupa-Krebzdak et al., 2018	Filtered water, organic whole soybeans, organic fair trade unrefined cane sugar, calcium carbonate, salt, organic fair trade vanilla flavor, vitamin A palmitate, gellan gum, riboflavin (vitamin B2), vitamin B12.	46	4.58	2.92	1.67	_	125.00	_	_	_	USDA Food Composition Database.
Chalupa-Krebzdak et al., 2018	Filtered water, whole organic soybeans, evaporated organic cane juice, calcium carbonate, organic natural flavours, sea salt, xanthan gum, carrageenan, vitamin A palmitate, riboflavin (B2), vitamin D2, vitamin B12.	42	3.75	2.92	1.67	_	125.00	_	_	_	USDA Food Composition Database.
Decloedt et al., 2018	Water, soybeans (pealed), tricalcium phosphate (5.90%), monopotassium phosphate (acid regulator), salt, aromas, sugar, gellan gum.	39	2.50	3.00	1.80	0.50	120.00	_	_	44.00 ^a^	Nutritional values obtained from the label of the drinks that were purchased.
Ferreira, 2011	Water, soybeans (1:8 soy:water ratio).	29 ^b^	3.21 ^b^	3.39 ^b^	1.13 ^b^	_	_	_	_	_	Beverage prepared and analyzed for the study.
Ferreira, 2011	Water, soybeans (1:10 soy: water ratio).	28 ^b^	1.41 ^b^	1.62 ^b^	0.98 ^b^	_	_	_	_	_	Beverage prepared and analyzed for the study.
Ferreira, 2011	Water, soybeans (1:12 soy: water ratio).	16 ^b^	1.18 ^b^	1.55 ^b^	0.51 ^b^	_	_	_	_	_	Beverage prepared and analyzed for the study.
Hajirostamloo, 2009	Water, soybean.	33 ^b^	1.86 ^b^	2.82 ^b^	1.96 ^b^	1.33 ^b^	4.11 ^b^	0.59 ^b^	_	_	Beverage prepared and analyzed for the stud.y
Martínez-Padilla et al., 2020	Water, shelled soybean (7.20%).	35	0.10	3.70	2.10	_	_	_	_	_	Nutritional values obtained from the label. Brand name: Naturli.
Nti et al., 2016	Water, soybeans, salt (0.20%).	50 ^b^	6.85 ^b^	2.60 ^b^	1.62 ^b^	_	19.00 ^b^	0.51 ^b^	22.59 ^b^	2.57 ^b^	Beverage prepared and analyzed for the study.
Scholz-Ahrens et al., 2020	Water, dehulled soyabeans (7.20%), apple concentrate (3.30%), algae Lithothamnium calcareum (0.40%), sea salt.	46 ^b^	2.46 ^b^	3.80 ^b^	2.16 ^b^	_	123.24 ^b^	_	_	_	Nutritional values obtained from the label. Brand name: Provamel.
Scholz-Ahrens et al., 2020	Water, raw cane sugar, dehulled soyabeans (5.80%), fat reduced cocoa (1.30%), chocolate (1.10%), sea salt.	69 ^b^	8.32 ^b^	3.49 ^b^	2.26 ^b^	_	ND	_	_	_	Nutritional values obtained from the label. Brand name: Provamel.
Uliana and Venturini Filho, 2010	Water, soybean.	33 ^b^	2.11 ^b^	2.82 ^b^	1.43 ^b^	_	_	_	_	_	Beverage prepared and analyzed for the study.
Uliana and Venturini Filho, 2010	Water, soybean.	31 ^b^	1.80 ^b^	2.88 ^b^	1.39 ^b^	_	_	_	_	_	Beverage prepared and analyzed for the study.
Uliana and Venturini Filho, 2010	Water, soybean.	31 ^b^	1.85 ^b^	2.82 ^b^	1.34 ^b^	_	_	_	_	_	Beverage prepared and analyzed for the study.
**Spelt-based beverage**											
Scholz-Ahrens et al., 2020	Water, spelt (7.00%), oat (6.00%), maltodextrin, barley malt (4.00%), high-oleic sunflower oil, cocoa (1.00%), algae Lithothamnion maerl (0.50%), sea salt, locust bean gum powder.	83 ^b^	15.41 ^b^	1.13 ^b^	1.85 ^b^	_	123.24 ^b^	_	_	_	Nutritional values obtained from the label. Brand name: Swiss cereal drink.
**Sunflower seed-based beverage**											
Blum et al., 2016	Water (distilled), germinated sunflower seeds.	21 ^b^	0.00 ^b^	0.77 ^b^	2.00 ^b^	1.20 ^b^	0.16 ^b^	_	_	1.88 ^b^	Beverage prepared and analyzed for the study.
***Tucumã* almond-based beverage**											
Silva et al., 2015	Water, *tucumã* almond flour.	6 ^b^	0.44 ^b^	0.06 ^b^	0.45 ^b^	_	_	_	_	_	Beverage prepared (extraction temperature of 55 °C) and analyzed for the study.
Silva et al., 2015	Water, *tucumã* almond flour.	7 ^b^	0.47 ^b^	0.07 ^b^	0.51 ^b^	_	_	_	_	_	Beverage prepared (extraction temperature of 75 °C) and analyzed for the study.
Silva et al., 2015	Water, *tucumã* almond flour.	10 ^b^	0.38 ^b^	0.07 ^b^	0.92 ^b^	_	_	_	_	_	Beverage prepared (extraction temperature of 100 °C) and analyzed for the study.
**Yam-based beverage**											
Araújo, 2015	Water, yam.	10 ^b^	2.40 ^b^	0.17 ^b^	0.01 ^b^	0.18 ^b^	3.59 ^b^	0.06 ^b^	_	0.82 ^b^	Beverage prepared for the study. Nutritional values taken from the TACO and the IBGE food composition table.

CHO: carbohydrate; Ca: calcium; Fe: iron; Mg: magnesium; Na: sodium; ND: not declared; kGy: the unit dose of ionizing irradiation. Standardization of energy values to integers. ^a^ Sodium value obtained from the salt value (1 g of salt is equivalent to 400 mg of sodium). ^b^ Values converted to standard serving size (100 mL).

## Data Availability

This study did not report any data.

## Appendix A. Search Terms Used to Perform the Literature Search

English	Milk alternative; Milk substitute; Plant-based milk; Plant-based beverage; Plant-based drink; Plant-based alternative; Milk analog; Non-dairy milk; Non-dairy beverages; Non-dairy alternative; Nondairy beverage; Plant milk; Vegan milk; Vegetable milk; Plant-based dairy
Portuguese	*Extrato vegetal; Extrato hidrossolúvel; Bebida à base de*

## Appendix B. Table of Authors, Year of Publication and Country of Origin of the Selected Studies According to the Inclusion and Exclusion Criteria

**Authors**	**Year of Publication**	**Country of Origin**
Abrão [30]	2019	Brazil
Alozie; Udofia [58]	2015	Nigeria
Andrade [31]	2018	Brazil
Araújo [41]	2015	Brazil
Barros [43]	2012	Brazil
Barros [42]	2016	Brazil
Barros; Venturini Filho [44]	2016	Brazil
Blum et al. [45]	2016	Brazil
Carvalho et al. [46]	2011	Brazil
Chalupa-Krebzdak et al. [23]	2018	Canada
Decloedt et al. [52]	2018	Belgium
Demoliner [47]	2019	Brazil
Ferreira [48]	2011	Brazil
Hajirostamloo [56]	2009	Iran
Holanda [32]	2017	Brazil
Jeske [57]	2018	Ireland
Karimidastjerd; Kilic-Akyilmaz [60]	2021	Turkey
Lima et al. [33]	2020	Brazil
Manassero et al. [51]	2020	Argentina
Manzoor et al. [59]	2017	Pakistan
Martínez-Padilla et al. [53]	2020	Denmark
Meeshi et al. [49]	2014	India
Nti et al. [55]	2016	Ghana
Ravindran; RadhaiSri [50]	2020	India
Reis [34]	2019	Brazil
Scholz-Ahrens et al. [54]	2020	Germany
Silva et al. [35]	2015	Brazil
Silva [36]	2018	Brazil
Storck; Montagner [37]	2020	Brazil
Uliana; Venturini Filho [38]	2010	Brazil
Vieira [39]	2013	Brazil
Vieira [40]	2017	Brazil
